# Clinical indications for total abdominal hysterectomy among women seen in Dar es Salaam regional referral hospitals, Tanzania: a prospective, observational hospital-based study

**DOI:** 10.11604/pamj.2021.38.10.17695

**Published:** 2021-01-06

**Authors:** Godfrey Jacob Chale, Rashid Mohammed Salim, Kelvin Melkizedeck Leshabari

**Affiliations:** 1 Department of Obstetrics and Gynaecology, International Medical and Technological University, Dar es Salaam, Tanzania,; 2 Clinical Research, H3 Research Unit of I-Katch Technology Limited, Dar es Salaam, Tanzania,; 3 Founder Trustee, Ultimate Family Healthcare Trust3, Dar es Salaam, Tanzania

**Keywords:** Hysterectomy, surgery, prospective, Dar es Salaam, Tanzania

## Abstract

Total abdominal hysterectomy is among the commonest gynaecologic surgeries observed in Africa. However, there exists a gap in published data to support this hypothesis. Information on hysterectomies reported from sub-Saharan Africa reflects mostly obstetric indications. A prospective hospital-based study was conducted in Dar es Salaam, Tanzania from March-October 2017. Women attending the facilities with clinical conditions necessitating abdominal hysterectomies were the target population. Each woman was followed from the time of planning for surgery until at most 72-hours post-surgery or discharge from the wards whichever came first. Continuous variables were summarized using median (with corresponding inter-quartile range). Categorical variables were summarized using frequency (%). Data outputs were created using SAS version 9.4. Verbal informal consent was sought from each individual prior to inclusion to this study. We recruited and prospectively followed-up 107 patients. Median age of participants was 42 (IQR: 37-47) years. Uterine leiomyoma (84.1%) was the leading indication for hysterectomy. Only about a third (30.8%) of followed-up women had provisional diagnoses at the time of surgery. None of the study participants reported receipt for confirmatory histological findings of her uterus up to the hospital discharge time post-surgery.

## Introduction

Hysterectomy is the commonest gynaecologic surgery reported in various parts of the world [1-3]. It is second only to caesarean section as the commonest surgery among women in the United States [4]. Data from other geographical places, including Africa, reflect the same trend [5,6]. However, caesarean section is an obstetric procedure, leaving hysterectomy the commonest surgery, among women in non-gravid state. There is palpable evidence that shows hysterectomy rates to differ according to patient-related, physician-based as well as geographic factors [5-7]. For instance, hysterectomy has been reported to be commoner among blacks than whites, [5-8] but the observation is confounded by clinical indications necessitating the surgical procedure. Thus, clinical indications necessitating hysterectomy need to be assessed regularly, especially in sub-Saharan Africa, for both clinical quality improvements as well as for standards regulation. The aim of this specific study trajectory was to assess clinical indications among women admitted for total abdominal hysterectomy at Dar es Salaam regional referral hospitals in Tanzania.

Hysterectomy can be performed for benign or malignant indications. Moreover, it can be performed as an emergency or elective surgery. However, removal of the uterus renders the woman without the inborn safe house for her future developing foetus (es). Thus, hysterectomy is justifiable upon weighing the risk-to-benefit ratio given the client´s underlying clinical presentation. Moreover, case-fatality rates of hysterectomy are higher in sub-Saharan Africa than the rest of the world [9]. At present, there is scarcity of data on clinical indications among women undergoing total abdominal hysterectomy in Tanzania. The available published evidence are findings on emergency postpartum hysterectomy [10]. The quest for indications of total abdominal hysterectomy in Tanzania performed on an elective basis is widening. We aimed to answer this call using a prospective facility-based observational study by design.

## Methods

A prospective facility-based follow-up study was done at Amana, Mwananyamala and Temeke municipal referral hospitals from March to October 2017. The three hospitals are the public referral facilities for Dar es Salaam residents respectively. Clients attending to the three municipal hospitals are ordinary Tanzanians with rich demographic mixture ranging from Bantu to Afro-Arabic people. Afro-Arabic people constitute a growing population segment in today´s Tanzania and are mostly from the coastal areas of Tanzania, including the islands of Unguja and Pemba. Women admitted for total abdominal hysterectomy at gynaecology wards were the target population. Each woman was followed-up from the time of admission to at most 72 hours or the time of discharge from the hospital post-surgery.

Data collection was via a pre-designed clinical sheet. Pre-operative clinical work-up was also recorded from each participant by either direct measurement or indirectly via a system-based follow-up. Specifically, pre-operative clinical information on indications for the planned surgical procedure forms the base for the findings in this article. Data collected from clinical sheets were immediately triple entered into the pre-designed Epi-info software template, cleaned and stored until analysis time. Exploratory data analysis was also done. It consisted mainly of univariate analysis for selected variables of interest. Continuous data were summarized using median (with corresponding inter-quartile range) while categorical variables were summarized using frequency (with relative frequency expressed as %). SAS statistical software version 9.4 gave the statistical outputs from the final coded dataset.

It was transferred into MS-Excel sheet from the original Epi-info file. The study protocol received institutional ethical clearance from the ethics committee of the International Medical and Technological University. Furthermore, permission to conduct the study at each municipal referral facility was obtained from offices of respective municipal officers of health as well as from the medical directors of Amana, Mwananyamala and Temeke regional referral hospitals. A verbal informed consent was sought from each woman before inclusion into the study.

## Results

The study managed to follow-up 107 clients between March-October 2017. Respondents were largely in their 5^th^ decade of life with the median age of 42 (IQR: 37-47) years. Out of 107 clients, 106 (99.1%) had their clinical diagnosis known at the time of surgery. Clinical indications for total abdominal hysterectomy made pre-operatively are as shown in Table 1 below.

**Table 1:** clinical indications for total abdominal hysterectomy among women admitted in Dar es Salaam regional referral hospitals (March - October 2017)

Reported surgical indications	Frequency	Proportion by percentage (%)
Uterine myoma	90	84.1
Chronic pelvic pains	6	5.6
Abnormal uterine bleeding	3	2.8
Ovarian tumour	2	1.86
CIN III	2	1.86
Endometrial carcinoma	1	0.93
Endometriosis	1	0.93
Uterine myoma and ovarian cysts	1	0.93
Uterine prolapsed	1	0.93

Moreover, out of 107 women followed-up, 73 (68.9%) had their pre-operative diagnoses confirmed prior to surgery. The rest (n=33, 30.8%) in exception of one had provisional diagnoses prior to surgery. One client whose diagnosis of uterine leiomyomata was made had dubious pre-operative clinical work-up. Authors decided to separate that client as a special independent case from the approved dichotomy (i.e. provisional vs. confirmatory diagnosis) during analysis. Most clients with provisional diagnoses at the time of surgery were reported to be suffering from abnormal uterine bleeding and chronic pelvic pains. Also, of the three clients whose indications for surgery were pelvic peritonitis, one had a decision for surgery made on a provisional ground, due to logistical challenges in laboratory and imaging units at the time of her admission.

Likewise, we noted that none of our followed-up women had liver and kidney function tests reported/documented prior to surgery. Similarly, for all women whose diagnoses were uterine leiomyomata, decision for surgery was made after imaging findings confirming the provisional diagnosis. None of these women reported to had had received histological results confirming their uteri removal was indeed attributed to uterine leiomyomata up to and including the time of discharge from the hospital post-hysterectomy.

## Discussion

Uterine leiomyomata was the leading indication (84.1%) for hysterectomy observed in our study settings. Our finding is similar to previous published studies in sub-Saharan Africa [6]. For some yet debatable reasons, uterine leiomyomata is evident to be commoner among black women than in women of other races [5-8]. However, the fact that in our study, on average about four-out-of-every five women, whose uteri were surgically removed, benign uterine growth was the indication, warrants attention. It is no doubt that at least half of our study population was still in their reproductive age. Therefore, there was a compelling indication for preservation of their uteri. Besides, on an account of psychological, as well as traumatic stress, that is associated with removal of parts of human bodies among clients further necessitates a second look on the current statistic. We do believe that a significant proportion of these women could have their uteri preserved should other management options considered.

At this point, it is also important to remember studies reporting hysterectomies from sub-Saharan Africa to be dominated by indications related to emergency obstetrics. Available plausible answers have evidence from studies done somewhere else in the developing world [6,9,10]. They reported maternal life-saving to be the sole determinant for emergency hysterectomy on obstetric grounds, solely due to complications associated with child births [9,10]. In contrast, we do believe our current article to additionally contribute towards building published evidence for gynaecologic indications of hysterectomies in sub-Saharan Africa.

Moreover, there are a number of design issues worth acknowledgement when one wants to analyse, interpret and compare our findings, especially among clinical researchers and policy makers. First, our study unlike others in similar settings was prospective by design. Majority of studies we retrieved that report indications for hysterectomies in sub-Saharan Africa were retrospective by design [6,9,10]. Retrospective observational findings though as important and in some cases (e.g. in observation of rare events/outcomes) more efficient than prospective studies, are dangerous for comparison in this specific aspect. This is because retrospective studies are largely dependent on recorded or recalled information in space and time. Thus, despite design issues associated with observational nature, our study findings benefits readers on an account of information collection at real time using a prospective design.

Another aspect worth recognition, on the basis of design issues, lies on the observational nature of our study findings. The ideal situation to answer our research question was to conduct an interventional or probably an audit study. We still do believe that our findings were reflective of routine gynaecological surgical activities. Likewise, the observation that none of women followed-up in our study had liver and kidney function tests recorded as baseline clinical work-up prior to surgery is thought provoking and worth thorough appraisal by responsible bodies. Again, we speculate multi-factorial causal pathways for this finding. For instance, just as in most cases, that demanded histological analyses, for diagnoses of possible malignancies, samples had to be sent to Muhimbili National Hospital that is located kilometers away from the studied hospitals; it is also probable that the laboratory units in our study settings were devoid of resources to perform liver and kidney function tests. Otherwise, should the tests have been done but not reported, it is also as risky and a dangerous endeavour. We do believe clinical audit to be a solution to this adverse observation.

## Conclusion

Uterine leiomyoma was reported as the leading condition necessitating hysterectomy in this study population. Malignant indications for hysterectomy were rarely reported in our study population.

### What is known about this topic

- Hysterectomies are common obstetric surgical procedures in sub-Saharan Africa;
- Hysterectomies are commoner among African than Caucasian women;
- Emergency hysterectomies have a potential to save maternal life if performed on appropriate indications and on a timely basis.

### What this study adds

- Total abdominal hysterectomy is prevalent even among women in their reproductive age in Dar es Salaam;
- There appears to be missing evidence of histological support on decision for total abdominal hysterectomy done to Dar es Salaam women at the study settings during the study time.
